# Supplementary material for: Rosmarinic Acid, as an NHE1 Activator, Decreases Skin Surface pH and Improves the Skin Barrier Function
Source: Int J Mol Sci. 2022 Mar 31;23(7):3910. doi: 10.3390/ijms23073910 (PMC8999067; doi:10.3390/ijms23073910)
Supplement: Supplementary file 1 [file ijms-23-03910-s001.zip › ijms-1622916-supplementary.pdf]

Table S1. Summary of the calibration curve, linear range, limit of detection (LOD), and limit of quantification (LOQ) for SC lipids

Type	Species	Dynamic range (pmol)	Slope	r^2	Y intercept	LOD[1] (pmol)	LOQ[2] (pmol)
Ceramide NP	N-16:0 Phytosphingosine	0.5–500	0.0079	0.9987	0.0125	0.1051	0.3186
	N-18:0 Phytosphingosine	0.5–500	0.0071	0.9971	0.0117	0.1634	0.4951
	N-24:0 Phytosphingosine	0.5–500	0.0057	0.9989	0.0103	0.1458	0.4419
Free fatty acid	C16	0.5–500	0.0062	0.9968	0.0222	0.2097	0.6348
	C18	0.5–500	0.0055	0.9955	0.0150	0.2113	0.6401
	C24	0.5–500	0.0054	0.9979	0.0226	0.2341	0.7098
Cholesterol	Cholesterol	2–500	0.0021	0.9962	0.0049	0.5598	1.6975

[1]LOD = 3.3×σ/S

[2]LOQ = 10×σ/S

(σ = Standard deviation of the response, S = The slope of the calibration curve.)

Table S2. Accuracy and precision of SC lipid analysis

Type	Spike amount (pmol)	Precision (RSD[1], %)	Accuracy (Accuracy[2], %)
	0.7	11.22	88.06 ± 11.88
N-16:0 Phytosphingosine	5	5.08	101.20 ± 3.66
	400	1.37	97.34 ± 1.02
	0.7	14.91	89.04 ± 13.49
N-18:0 Phytosphingosine	5	6.81	100.64 ± 3.73
	400	2.39	103.73 ± 1.19
	0.7	15.26	87.12 ± 14.59
N-24:0 Phytosphingosine	5	7.40	103.11 ± 4.08
	400	2.08	99.78 ± 1.11
	0.7	14.67	89.75 ± 8.57
C16:0 Free fatty acid	5	5.44	104.11 ± 4.28
	400	2.61	100.57 ± 1.58
	0.7	13.03	87.37 ± 9.11
C18:0 Free fatty acid	5	3.30	94.42 ± 2.39
	400	1.87	100.91 ± 1.74
	0.7	10.88	89.10 ± 9.54
C24:0 Free fatty acid	5	3.28	94.40 ± 3.95
	400	2.52	102.09 ± 2.17
	3	8.95	89.79 ± 7.47
Cholesterol	10	2.33	99.96 ± 2.01
	400	1.12	100.55 ± 1.03

[1]RSD (%) = (standard deviation of the concentration/mean concentration) × 100

[2]Accuracy (%) = (calculated concentration/theoretical concentration) × 100
